# Effect of pH and buffer on substrate binding and catalysis by *cis*-aconitate decarboxylase

**DOI:** 10.1038/s41598-025-89341-1

**Published:** 2025-02-11

**Authors:** Mingming Zhao, Chutao Chen, Wulf Blankenfeldt, Frank Pessler, Konrad Büssow

**Affiliations:** 1 Department of Structure and Function of Proteins, Helmholtz Centre for Infection Research, Braunschweig, Germany; 2 Research Group Biomarkers for Infectious Diseases, TWINCORE Centre for Experimental and Clinical Infection Research, a Joint Venture Between Hannover Medical School and the Helmholtz Centre for Infection Research, Braunschweig, Germany; 3 Institute for Biochemistry, Biotechnology and Bioinformatics, Technische Universität Braunschweig, Braunschweig, Germany; 4 Centre for Individualised Infection Medicine, Hannover, Germany

**Keywords:** Biochemistry, Biocatalysis, Biophysical chemistry, Enzyme mechanisms, Enzymes, Innate immune cells, Innate immunity

## Abstract

*cis*-Aconitate decarboxylase (ACOD1, CAD, IRG1) catalyses the synthesis of itaconic acid in activated myeloid cells such as macrophages. Several histidine residues in the active site bind the substrate and enable the decarboxylation reaction. The in vitro activity of ACOD1 enzymes is commonly determined by incubation with substrate, followed by HPLC measurement of itaconic acid production. Phosphate buffers have often been used for this assay. However, the influence of buffer type on enzyme activity has not been investigated. Here, the effect of buffer and pH on enzyme kinetics of human and mouse ACOD1 and *Aspergillus terreus* CAD was investigated. It was found that high concentrations of phosphate inhibit the three enzymes. An alternative buffer was selected and the assay was adapted to the 96-well microtitre plate format for increased throughput. Enzyme kinetics were determined in the pH range of 5.5–8.25. A strong increase of *K*_M_ values was observed between the physiologically relevant pH values 7.5 and 8.25. The data indicate that more than one histidine residue needs to be protonated in the active site for binding the substrate.

**Supplementary Information:**

The online version contains supplementary material available at 10.1038/s41598-025-89341-1.

## Introduction

**Fig. 1:**
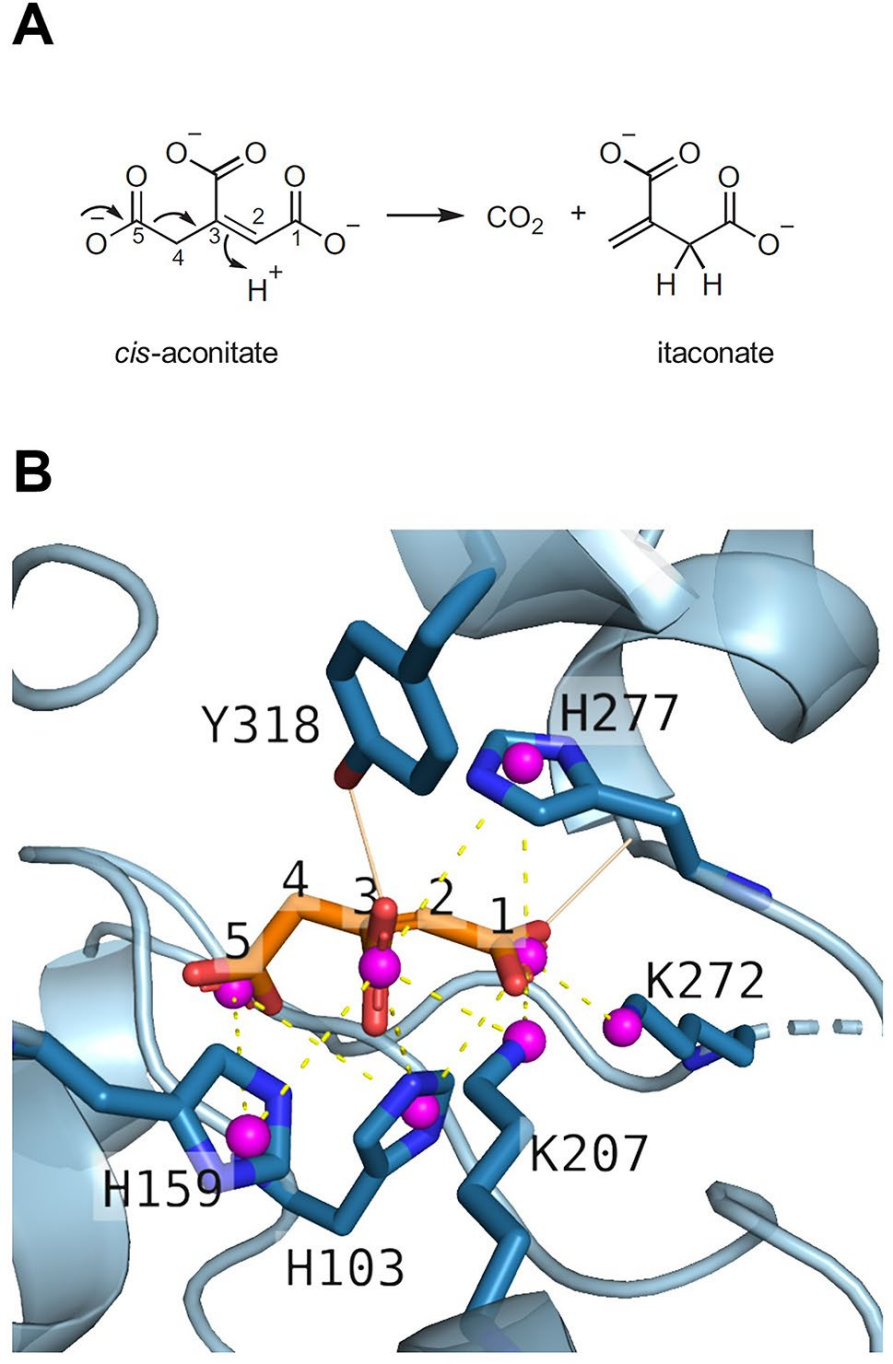
ACOD1 reaction and substrate complex. (**A**) The formation of itaconate catalysed by ACOD1. Bentley and Thiessen^9^ showed that ACOD1 removes the C5 carboxyl group and that a proton from the solvent is added to position C2. (**B**) Model of the complex of human ACOD1 with *cis*-aconitate. The active site of the hACOD1 structure (PDB ID code 6R6U) is shown with the putative conformation of the substrate *cis*-aconitate described in Chen et al.^6^. Active site residues are shown in blue and *cis*-aconitate in orange. Hydrogen bonds to Tyr318 and the main chain (solid yellow lines) and electrostatic interactions (dashed yellow lines) were predicted by the Protein Ligand Interaction Profiler (PLIP)^32,33^, assuming protonation of all histidines. Charge centres are represented by spheres in magenta. Hydrogen bonds between groups with electrostatic interactions are not shown.

*cis*-Aconitate decarboxylase catalyses the conversion of the tricarboxylic acid (TCA) cycle intermediate *cis*-aconitate to itaconate during activation of myeloid-derived cells (Fig. 1A)^1^. Work in a variety of experimental models, as well as human clinical data, have revealed itaconate as a multifaceted compound that can exert antibacterial, antiviral, anti-oxidative, cytoprotective, and immunomodulatory effects^2^. With respect to the latter, accumulating evidence has identified important pro-tumorigenic functions of ACOD1. Its expression in tumour tissue correlates with a poor clinical prognosis in a variety of cancers, likely due to immunosuppressive effects that reduce anti-tumor activity of CD8+ T cells in the tumour microenvironment^3,4^. In addition, direct (tumour-intrinsic) neoplastic effects are plausible in tumours where cancer cells themselves express ACOD1^5^. ACOD1 has thus emerged as a novel treatment target in oncology, necessitating the search for ACOD1 inhibitors that prevent itaconate synthesis.

We previously elucidated the crystal structure of human and mouse ACOD1 and described a cell-free ACOD1 activity assay based on recombinant ACOD1 expressed in *E. coli*^6^. We subsequently used this assay to identify amino acid positions that determine the reduced activity of human ACOD1^7^. The assay was also used to screen for potential ACOD1 inhibitors that resembled the transition state of the enzymatic reaction and identified citraconic acid (a naturally occurring isomer of itaconic acid) as a competitive inhibitor, which may serve as scaffold for further pharmacologic optimization^8^.

The effect of pH on substrate binding and catalytic rate of ACOD1 enzymes has not been investigated, nor has the potential impact of specific buffers been explored. Buffer molecules may interact with enzymes and thereby affect their activity. The buffer’s pH can change enzyme activity in different ways. Extreme pH values usually cause protein denaturation. Moderate pH changes can modify enzyme activity if they affect the protonation of critical groups of the enzyme, the substrate or a cofactor. In the case of ACOD1, pH influences the protonation of the histidine residues in the active site (Fig. 1B). Protonation of these residues could be required to neutralize the negative charges of the *cis*-aconitate substrate.

## Results

**Fig. 2:**
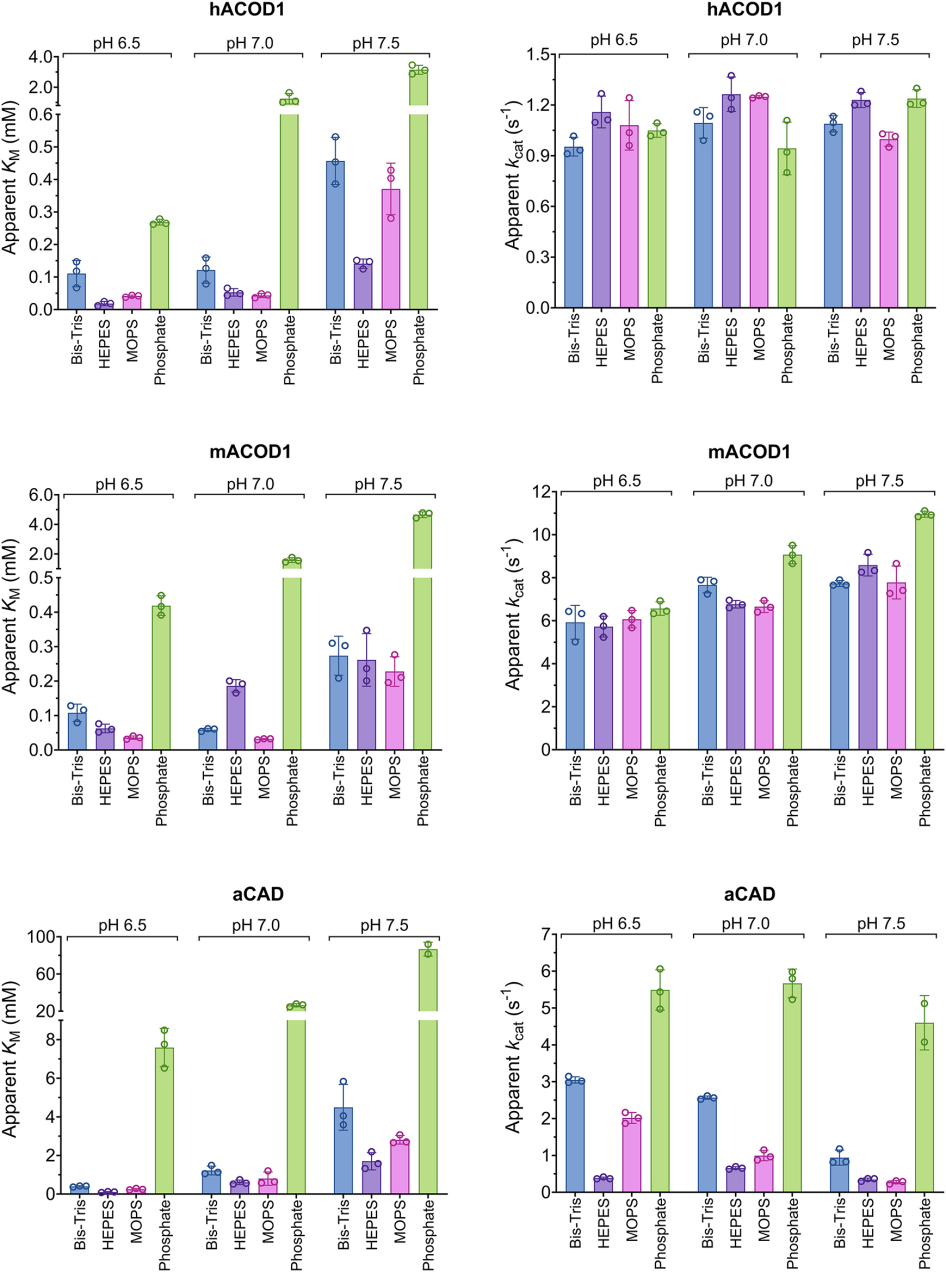
ACOD1 activity in phosphate and alternative buffers. The apparent *K*_M_ and *k*_cat_ values of hACOD1, mACOD1 and aCAD using 200 mM Bis-Tris, HEPES, MOPS and sodium phosphate buffer were compared at pH 6.5, 7.0 and 7.5. The corresponding Michaelis–Menten curves are shown in Fig. S1.

**Fig. 3:**
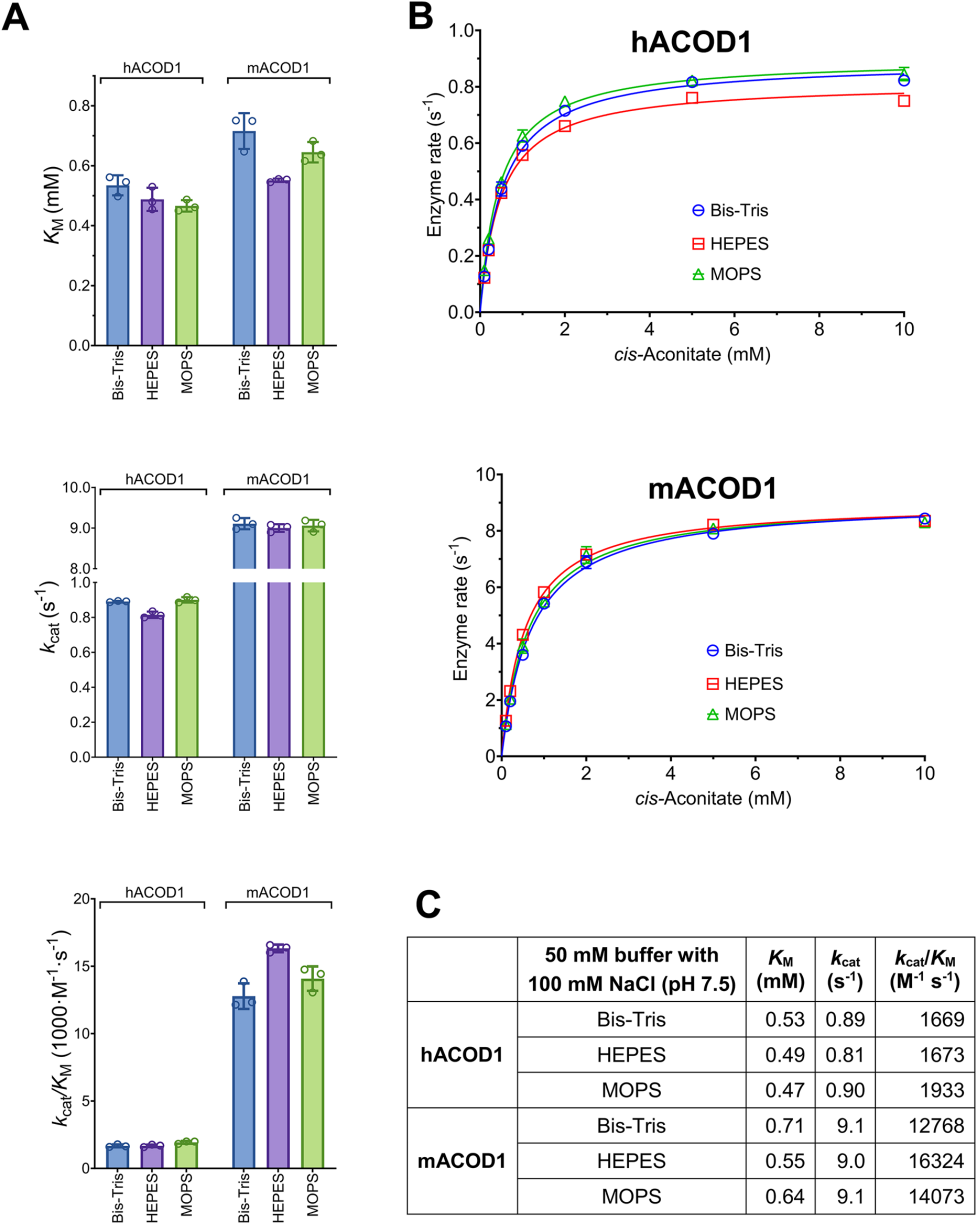
Comparison of Bis-Tris, MOPS and HEPES buffers. The enzyme kinetics of hACOD1 and mACOD1 were similar using 50 mM Bis-Tris, HEPES or MOPS buffers with 100 mM NaCl at pH 7.5. (**A**) Michaelis constants (*K*_M_), catalytic rate constants (*k*_cat_) and specificity constants (*k*_cat_/*K*_M_) of hACOD1 and mACOD1 in different buffers. (**B**) Raw data for the diagrams in A and table C.

In our previous ACOD1 assays, 125 µl of 0.2 M sodium-phosphate buffer, pH 6.5, was mixed with 25 µl enzyme and substrate solutions, resulting in 167 mM final phosphate concentration. This assay, originally reported by Bentley et al. for pH 5.6^9^ had been adapted to pH 6.3 by Dwiarti et al.^10^. ACOD1 assays have also been performed in HEPES buffer^11,12^. We investigated the influence of the buffer substance on enzyme activity. The 200 mM sodium phosphate buffer was replaced with MOPS, HEPES or Bis-Tris buffer at pH 6.5, 7.0 and 7.5 in assays of the human, mouse and *A. terreus* enzymes (Fig. 2, Fig. S1). The effect of temperature on pH was corrected by determining the buffers’ temperature coefficients (Fig. S2). A strong competitive inhibition of all three enzymes by 167 mM phosphate was noted at all three pH values. In further assays, 100 mM NaCl was added to the buffers to achieve a similar ionic strength independent of buffer substance and pH. Assays using 50 mM MOPS, HEPES or Bis-Tris buffers with 100 mM NaCl and pH 7.5 were performed and *K*_M_ and *k*_cat_ were essentially independent of buffer substance for the three enzymes (Fig. 3).

**Fig. 4:**
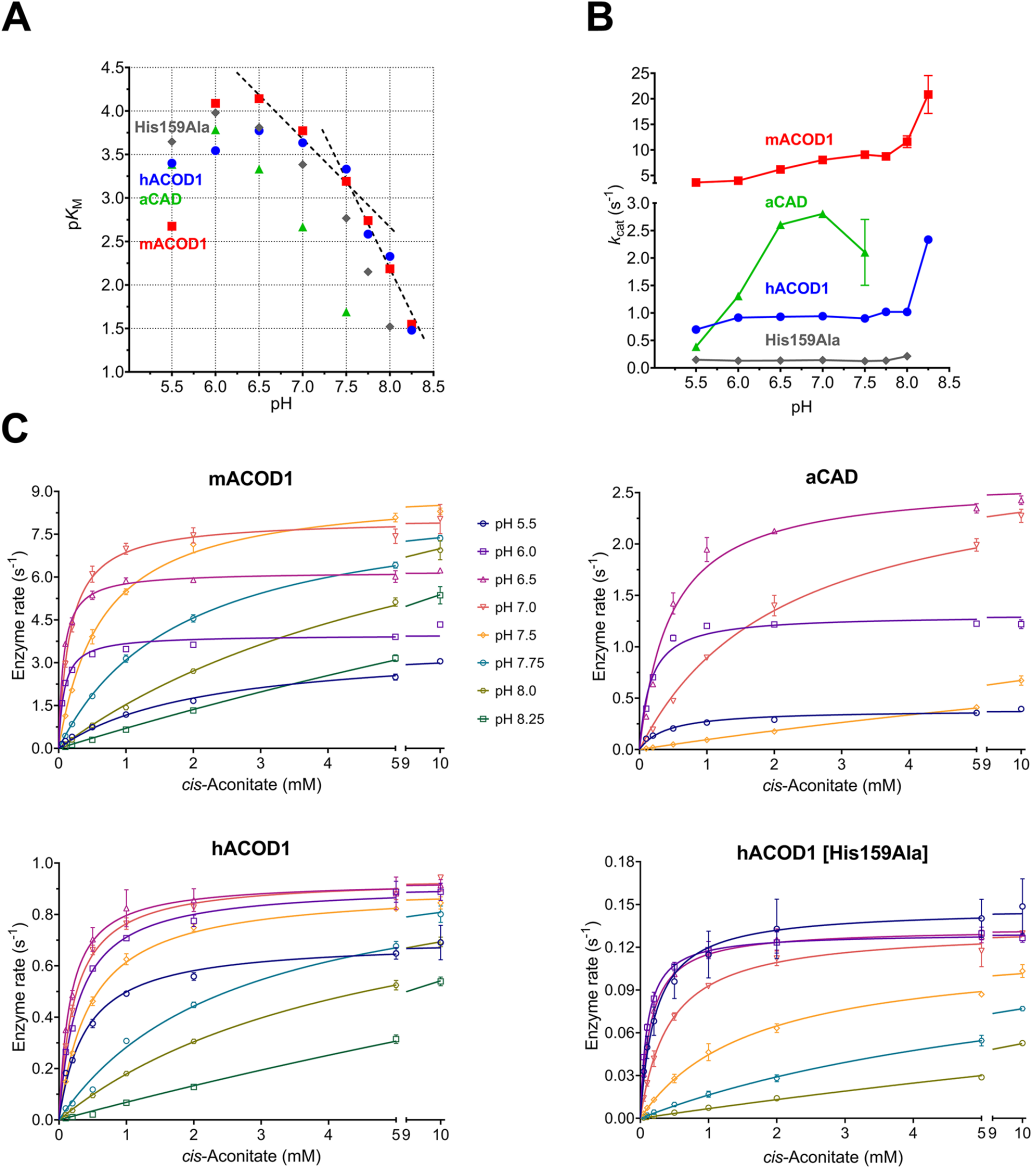
Effect of pH on enzyme activity. Enzyme kinetics of hACOD1, mACOD1, aCAD and the hACOD1 His159Ala mutant were measured at a pH range of 5.5–8.25. (**A**) The effect of pH on *K*_M_ of ACOD1, the His159Ala mutant and aCAD. p*K*_M_ = −log_10_(*K*_M_·M^−1^) was plotted over pH^13^. Slopes of −1 and −2 are indicated by dashed lines. (**B**) Effect of pH on *k*_cat_ of ACOD1 and aCAD. The catalytic constant *k*_cat_ of wild-type hACOD1, the His159Ala mutant and mACOD1 was essentially unchanged over pH 5.5–8.0. aCAD was inactive at pH > 7.5. (**C**) Michaelis–Menten diagrams of the data presented in (**A**) and (**B**) and Table 1.

We decided to use 50 mM MOPS buffer with 100 mM NaCl for further assays, since the p*K*_a_ of 7.0 at 37 °C of MOPS allows to use this buffer throughout a pH range of 5.5 to 8.5. The enzyme concentration was reduced to 0.2 µg per 150 µl assay, corresponding to approx. 27 nM monomer subunits of the homodimeric enzymes. This allows for assays with tight-binding inhibitors at low inhibitor concentrations without correcting for the reduction of free inhibitor concentration due to enzyme binding. Using the optimized assay, Michaelis constants (*K*_M_) and catalytic rate constants (*k*_cat_) for human ACOD1 (hACOD1), mouse ACOD1 (mACOD1) and *Aspergillus terreus* CAD (aCAD) were determined between pH 5.5 and 8.25 at pH intervals of 0.5 or 0.25 (Fig. 4; Table 1, Figs. S3–S5). *k*_cat_ of hACOD1 and mACOD1 was essentially unchanged over pH 5.5–8.0, whereas aCAD had the highest *k*_cat_ at a slightly acidic pH range of 6.5–7.0. For all three enzymes, the *K*_M_ values increased strongly from pH 7.0 to 8.25 by a factor of 20 or more.

**Table 1:** *K*_M_, *k*_cat_ and *k*_cat_/*K*_M_ values at pH 5.5–8.25.

50 mM MOPS, 100 mM NaCl	*K*_M_ (95% confidence interval) (mM)			
	hACOD1	mACOD1	aCAD	hACOD1 [His159Ala]
pH 5.5	0.40 (0.31–0.50)	2.1 (1.8–2.5)	0.41 (0.30–0.56)	0.23 (0.15–0.32)
pH 6.0	0.29 (0.25–0.33)	0.082 (0.065–0.10)	0.16 (0.13–0.21)	0.105 (0.093–0.118)
pH 6.5	0.17 (0.14–0.20)	0.072 (0.065–0.080)	0.47 (0.37–0.58)	0.155 (0.139–0.172)
pH 7.0	0.23 (0.21–0.26)	0.17 (0.15–0.19)	2.1 (1.9–2.5)	0.43 (0.37–0.50)
pH 7.5	0.47 (0.43–0.50)	0.64 (0.59–0.71)	20 (15–30)	1.65 (1.43–1.91)
pH 7.75	2.6 (2.3–3.0)	1.82 (1.73–1.91)		7.0 (6.0–8.1)
pH 8.0	4.7 (4.3–5.1)	6.5 (5.8–7.5)		30 (22–44)
pH 8.25	33 (24–49)	28 (21–42)		

50 mM MOPS, 100 mM NaCl	*k*_cat_ (95% confidence interval) (s^–1^)			
	hACOD1	mACOD1	aCAD	hACOD1 [His159Ala]
pH 5.5	0.70 (0.66–0.74)	3.6 (3.4–3.8)	0.38 (0.36–0.41)	0.147 (0.135–0.159)
pH 6.0	0.91 (0.89–0.94)	4.0 (3.8–4.1)	1.31 (1.24–1.38)	0.130 (0.127–0.133)
pH 6.5	0.93 (0.90–0.96)	6.2 (6.1–6.3)	2.6 (2.5–2.8)	0.133 (0.123–0.136)
pH 7.0	0.94 (0.92–0.96)	8.0 (7.8–8.2)	2.8 (2.7–3.0)	0.133 (0.128–0.138)
pH 7.5	0.90 (0.88–0.92)	9.1 (8.8–9.3)	2.0 (1.6–2.7)	0.118 (0.113–0.124)
pH 7.75	1.02 (0.96–1.08)	8.7 (8.6–8.9)		0.130 (0.121–0.142)
pH 8.0	1.02 (0.98–1.06)	12 (11–12)		0.21 (0.17–0.28)
pH 8.25	2.3 (1.8–3.2)	21 (16–28)		

50 mM MOPS, 100 mM NaCl	*k*_cat_/*K*_M_ (M^–1^ s^–1^)			
	hACOD1	mACOD1	aCAD	hACOD1 [His159Ala]
pH 5.5	1751	1714	935	639
pH 6.0	3197	48,543	7947	1238
pH 6.5	5525	85,499	594	858
pH 7.0	4060	47,467	1306	309
pH 7.5	1933	14,073	101	72
pH 7.75	392	4792		19
pH 8.0	217	1769		
pH 8.25	71	728		

The pH-dependence of *K*_M_ was analysed with a log-log plot of p*K*_M_ over pH according to M. Dixon^13^ (Fig. 4A). In this plot, a single residue that needs to be protonated for substrate binding would result in a curve with a slope of −1 at pH values greater than the residues p*K*_a_. The slope becomes steeper if two residues need to be protonated. The curves in Fig. 4A reach a slope of −2 at pH > 7.5, indicating that at least two residues with p*K*_a_ values below 7.5, presumably histidines, need to be protonated to allow substrate binding. There are three histidines in the active site that were predicted to form electrostatic interactions with *cis*-aconitate in ACOD1 (Fig. 1B). Nonlinear regression with equations corresponding to two or three titratable groups could fit the data equally well (Fig. S6).

His103 and His159 of hACOD1, but not His277, are conserved in aCAD. However, an aCAD structure predicted by AlphaFold2^14^ places His336 as third histidine into the active site, close to the position of His277 in hACOD1 (Fig. S7). Therefore, according to the AlphaFold prediction, aCAD also has three histidines that could interact with *cis*-aconitate.

The role of histidines 103, 159 and 277 in human ACOD1 was studied with alanine mutants described previously^6^. Only the His159Ala mutant had enzymatic activity. The mutation impaired both *K*_M_ and *k*_cat_ (Fig. 4; Table 1, Fig. S8). In the plot of p*K*_M_ over pH, the mutant’s profile was similar to the wild-type enzyme (Fig. 4A) with a slope of −2 at pH > 7.5, indicating that both of the remaining histidines, 103 and 277, need to be protonated for substrate binding.

In order to explore the physiological relevance of the above *K*_M_ values, we determined the *cis*-aconitate concentration in human macrophages as a relevant cell type. Enzyme activity depends on the accessible substrate concentration in relation to the *K*_M_ value. Human monocytic THP1 cells were differentiated with PMA to resemble macrophages, ACOD1 expression was induced by stimulation with LPS and IFNγ and *cis*-aconitate concentrations were measured after 24 h. *cis*-Aconitate was extracted from the cells and cellular protein was precipitated in parallel. *cis*-Aconitate amounts were determined by HPLC-MS/MS (high performance liquid chromatography-tandem mass spectrometry) and amounts of cellular protein were determined by a colorimetric assay. For calculation of intracellular *cis*-aconitate concentrations, cell numbers and volumes were deduced from the amounts of cellular protein (see Materials and methods). The concentration of *cis*-aconitate was 16 µM (0.38 pmol per µg cell protein) without stimulation and was somewhat lower after stimulation (Table 2).

**Table 2:** *cis*-Aconitate concentrations in THP1 cells.

Stimulation	*cis*-Aconitate (µM) ± SD	*cis*-Aconitate (pmol/µg cell protein) ± SD
None (*n* = 9)	16.3 ± 4.4	0.38 ± 0.10
LPS + IFNγ (*n* = 6)	11.8 ± 2.5	0.28 ± 0.06

## Discussion

Phosphate buffers of 167 mM concentration were found to inhibit ACOD1 activity in comparison to other buffers of the same concentration. Phosphate buffers contain doubly-charged hydrogen phosphate ions (HPO_4_^2−^) that increase the ionic strength four times more than an equivalent concentration of singly charged ions. It appears plausible that the higher ionic strength of the phosphate buffers has increased the ACOD1 *K*_M_ values by reducing the electrostatic forces between substrate ions and the active site. Moreover, a direct interaction of hydrogen phosphate ions with the positively charged residues of the active site could block access of the substrate. The buffer with 50 mM MOPS and 100 mM NaCl has a more moderate and less pH-dependent ionic strength over the pH range of 5.5–8.25 (ionic strength: 101–147 mM) and is better suited for studying the effect of pH on ACOD1 kinetics.

Phosphate had a twofold effect on *Aspergillus* CAD. Similar to the mammalian ACOD1 enzymes, aCAD was inhibited by phosphate in a competitive manner. In addition, aCAD was activated by phosphate at high substrate concentrations—its apparent *k*_cat_ was increased. This could be explained by an allosteric binding site where phosphate can bind independently of the substrate to accelerate the catalytic step.

A model of the enzyme-substrate complex has been proposed in which the substrate is bound via hydrogen bonds and electrostatic interactions between carboxyl groups of the substrate and histidine and lysine residues in the active site^6,8^. The protonated ACOD1 histidine 103 side chain (Fig. 1) delivers the proton for the decarboxylation reaction in this model. Increasingly basic pH values strongly increased the *K*_M_ values of the ACOD1 enzymes and aCAD, while *k*_cat_ was only affected moderately (Fig. 4). The pH dependence of the *K*_M_ values indicates that histidine residues that need to be protonated for substrate binding become deprotonated at basic pH^15^. A p*K*_M_-pH plot and nonlinear regression revealed that at least two residues in the active site with p*K*_a_ values below 7.5, presumably histidine residues, need to be protonated for formation of the enzyme-substrate complex (Fig. 4A, Fig. S7). This indicates that the active site histidines cannot bind the substrate by hydrogen bonds only, but that electrostatic interactions between protonated histidines and substrate carboxyl groups are required. Mutating histidine 103 or 277 to alanine abolished activity of hACOD1, while mutation of histidine 159 decreased it. The p*K*_M_-pH profile of the His159Ala mutant corresponded to the wild-type’s profile, indicating that substrate binding by the mutant requires protonation of the two remaining two histidines, 103 and 277, for balancing the negative charges of the substrate. It seems plausible therefore that the wild-type enzyme binds the substrate in a similar arrangement, with protonated residues His103 and His277 and unprotonated His159. *k*_cat_ did not change much with pH (Fig. 4B), which indicates that the histidine that donates the proton for the decarboxylation reaction (Fig. 1A) is always protonated in the enzyme-substrate complex, independent of pH.

Proton pumps of the respiratory chain cause a basic pH of the mitochondrial matrix that varies between pH 7.5 and 8.2, compared to a neutral pH in the mitochondrial intermembrane space^16^. It was often assumed that itaconic acid is synthesized in the mitochondrial matrix. However, a complex of ACOD1, the small GTPase Rab32 and leucine-rich repeat kinase 2 (LRRK2) was identified, localized at the outer mitochondrial membrane^17,18^, implying that itaconic acid is synthesized there. Correspondingly, an N-terminal mitochondrial targeting sequence (MTS) present in several Irg1-like proteins is missing in mammalian ACOD1 proteins^12^. The strong pH dependence of ACOD1 *K*_M_ values implies that the enzyme would be significantly more active at the outer membrane than in the mitochondrial matrix. For THP1 cells, an overall *cis*-aconitate concentration of 12 µM was measured upon stimulation with LPS and IFNγ (Table 2). At this substrate concentration and pH 7.0 and a corresponding *K*_M_ value of 230 µM, the catalytic activity of hACOD1 would be 5% of its maximum. Elevated *cis*-aconitate concentrations in close proximity to mitochondria would result in higher activity.

**Fig. 5:**
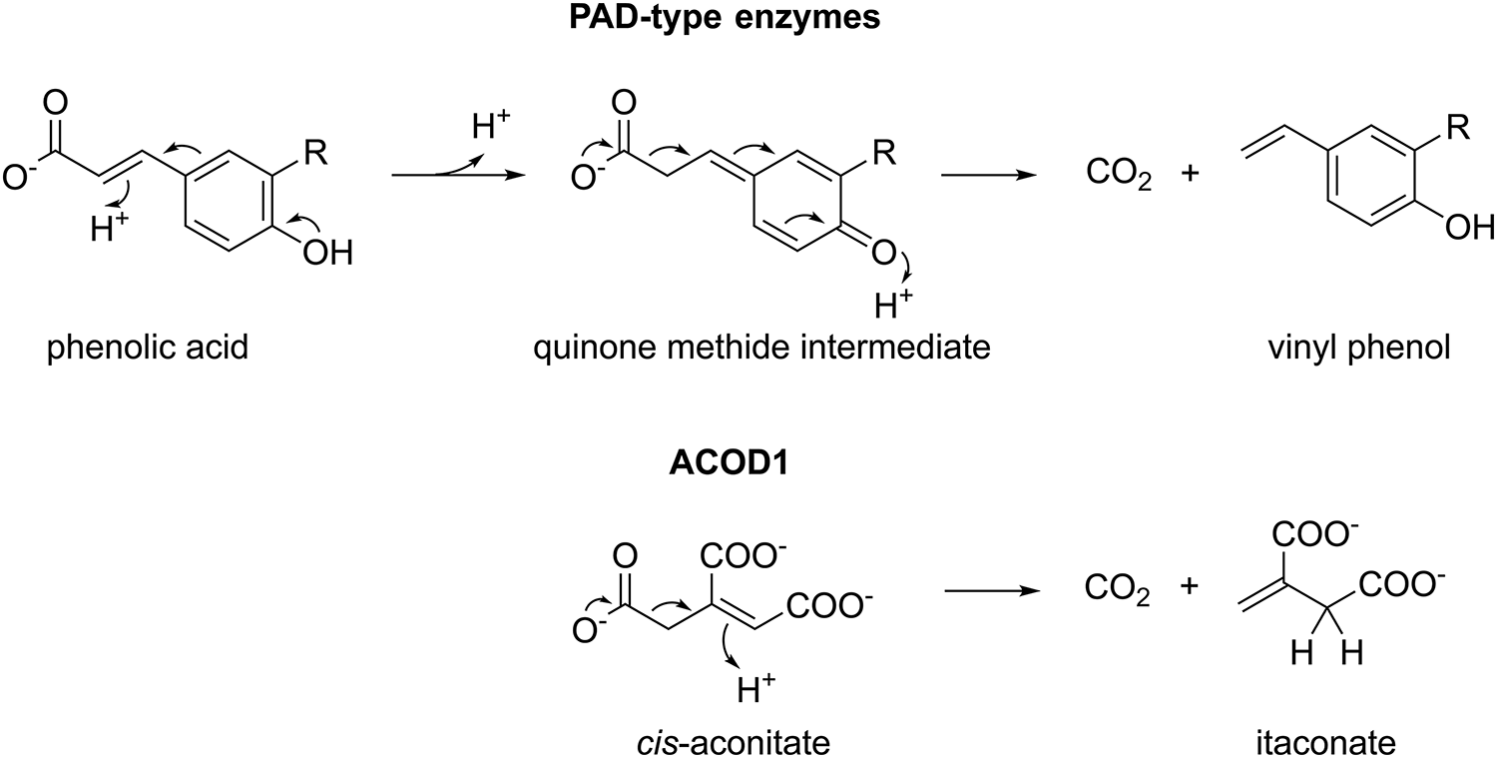
Reaction mechanisms of cofactor-independent decarboxylases. A simplified representation of the reaction mechanism of PAD-type enzymes^21^ and the reaction catalysed by ACOD1 are shown. Both are acid-base reactions that break the bond between the carboxylate leaving group and a methylene group by moving the bond electrons towards an adjacent double bond. R=H, OCH_3_ or OH.

Decarboxylation is a common metabolic reaction and most decarboxylases rely on metal ions or organic prosthetic groups as cofactors^19,20^. The reactions catalysed by cofactor-independent decarboxylases such as ACOD1 are often facilitated by reactive double bonds or aldehyde groups in the substrate molecules. The reaction mechanism of cofactor-independent phenolic acid decarboxylases (PAD) that convert phenolic acids such as *p*-coumaric acid to vinyl phenols bears similarity to ACOD1^21–24^. The reaction intermediates of these enzymes resemble *cis*-aconitate, as they also have a α methylene group (–CH_2_–) and β,γ double bond (Fig. 5). The mechanisms of decarboxylation of these intermediates and *cis*-aconitate might be comparable, since both reactions result in a terminal alkene group (=CH_2_).

The reaction mechanism of cofactor-independent decarboxylases typically involves a residue that donates a proton during the reaction. Studying the pH dependence of these enzymes can help identifying the proton-donating residue by determining its p*K*_a_ value. Malonate semialdehyde decarboxylase (MSAD) from *Pseudomonas pavonaceae* 170 catalyses the cofactor-independent decarboxylation of malonate semialdehyde (O=CH–CH_2_–COO^−^) to afford acetaldehyde and CO_2_. The protonated amino group of its N-terminal proline (Pro1) provides a proton to the substrate’s aldehyde oxygen in this reaction^25^. A p*K*_a_ value of 9.2 was determined for the amino group of Pro1 by pH titration and ^15^N NMR spectroscopy of the ^15^N-labeled enzyme^26^. This confirmed that the residue is protonated at the neutral pH of the enzyme assay. Arylmalonate decarboxylase (AMDase) from *Alcaligenes bronchisepticus* KU 1201 is another example of a cofactor-independent decarboxylase. It removes one of the two carboxyl groups of phenylmalonic acid and related compounds^27^. Using thiol reagents and mutagenesis, cysteine 188 was identified as a catalytic residue^28^. An activity-pH profile was obtained and a drastic decrease of activity was observed above pH 8 due to the deprotonation of Cys188^29^. In contrast to ACOD1, basic pH values affected only the *k*_cat_ of AMDase, but not the formation of the enzyme-substrate complex. This indicates that AMDase with deprotonated Cys188 can bind the substrate, but cannot carry out its decarboxylation, since this would require a proton from the Cys188 thiol group.

Optimization of the ACOD1 assay enabled determination of kinetic parameters over a wide pH range. Thereby the inhibition of the ACOD1 and CAD enzymes by phosphate and the importance of charged groups for the formation of the enzyme-substrate complex was revealed. The data support a model in which protonated histidine residues are required for substrate binding via electrostatic interactions. Information on the protonation status of the active site residues will be important for computer modelling for the enzymatic mechanism and for the rational design of ACOD1 inhibitors.

In summary, we have developed an optimized buffer for a cell-free ACOD1 enzyme assay. It should improve screening for ACOD1 inhibitors by providing data on inhibition that are closer to physiologic conditions than the originally used phosphate buffer. In addition, our findings about pH dependence of ACOD1 enzyme activity will prove important for designing studies on ACOD1 function in different subcellular compartments, organisms, and ecologic niches characterized by a range of pH values.

## Materials and methods

### Materials

di-Sodium hydrogen phosphate dihydrate (Na_2_HPO_4_·2 H_2_O), sodium dihydrogen phosphate monohydrate (NaH_2_PO_4_·H_2_O), 4-(2-hydroxyethyl)-1-piperazineethanesulfonic acid (HEPES) and 3-(N-morpholino) propanesulfonic acid (MOPS) were purchased from Carl Roth GmbH. Bis(2-hydroxyethyl)-amino-tris(hydroxymethyl)methane (Bis-Tris) and *cis*-aconitic acid were purchased from Sigma-Aldrich.

96-well 0.2 ml 8-Transformer Plates (Biozym Scientific GmbH, #712540) and BZO Seal Film (Biozym Scientific GmbH, #712350) were used for enzyme reaction. For subsequent loading into the HPLC autosampler, the plates were sealed with Sealing Tape (Thermofisher Scientific #232702). Plates were incubated in a 96-well PEQlab Universal PCR Thermal Cycler. The concentration of itaconic acid produced by enzymes was quantified using an HPLC system (LC-2050) from Shimadzu Corporation equipped with an Shodex RSpak DE-413 column. Enzyme solutions and substrates were added and mixed using 8-channel electronic Voyager pipettes from Integra.

### Expression and purification of enzymes

hACOD1 (aa 4–461), mACOD1 (aa 4–461), and aCAD (aa 12–490) proteins were produced in *Escherichia coli* and purified using clones CAD29, CAD39 and CAD16, as described previously^6,7^. A detailed protocol is available at protocols.io (10.17504/protocols.io.14egn2npyg5d/v2). Purified proteins were stored in GF buffer (10 mM HEPES, pH 7.4, 10% v/v glycerol, 150 mM NaCl, 1 mM TCEP) at −80 °C.

### Preparation of buffer solutions

The influence of the temperature on the p*K*_a_ of the buffers was corrected by determining the temperature coefficients ΔpH/°C. Each buffer was heated to around 37 °C and then gradually cooled while recording the pH values and corresponding temperature using a pH meter with thermometer. The temperature coefficient ΔpH/°C was determined by fitting a linear regression line to the pH vs. temperature data points. pH shifts were calculated based on this formula: ΔpH = ΔT × ΔpH/°C, where ΔT is the temperature difference between the preparation of the buffer and the assay temperature of 37 °C. The pH was adjusted using either HCl (Bis-Tris) or NaOH (HEPES and MOPS). For example, Bis-Tris buffer was adjusted to pH 7.76 at 20 °C to achieve pH 7.50 at 37 °C.

In this study, 200 mM sodium phosphate buffer solutions at specific pH values (6.5, 7.0, and 7.5) were prepared by mixing NaH_2_PO_4_ and Na_2_HPO_4_ solutions. Additionally, 200 mM and 50 mM Bis-Tris, HEPES, and MOPS buffer solutions with 100 mM NaCl were prepared and adjusted to various pH values (5.5, 6.0, 6.5, 7.0, 7.5, 7.75, 8.0, and 8.25) using the temperature-correction procedure described above.

*cis*-Aconitate was dissolved in water and neutralized with NaOH and stored at 450 mM at −80 °C. The original stock was diluted in water for the enzyme assays.

### Enzyme assay in 96-well plates

Reactions and assays were performed in 96-well plates with a total reaction volume of 150 µl. Except for the enzyme dilution, all steps were performed using 8-channel electronic pipettes. Proteins were diluted to 1 mg/ml in GF buffer, and exact concentrations were measured by spectrophotometry at 280 nm. The enzymes were further diluted to 0.2 µg enzyme per 140 µl in the reaction buffer. Subsequently, 10 µl of each substrate stock was dispensed into the bottom of each well using an 8-channel Voyager pipette with a volume range of 0.5–12.5 µl. Following this, 140 µl of enzyme solution was added to each well using an 8-channel pipette and starting with the lowest substrate concentrations. The reactions were mixed directly using the pipette’s mixing program, set to a 50 µl mixing volume and 4 mixing cycles. All of the above operations were performed on ice. The final enzyme concentration was 1.33 ng/µl. The 96-well plate was sealed with BZO Seal Film and centrifuged briefly. Before this, the PCR cycler lid and block were preheated to 95 °C and 37 °C, respectively. The 96 well plate was placed in the PCR cycler and incubated at 37 °C for 10 min (mACOD1) or 100 min (hACOD1 and aCAD), followed by 3 min at 95 °C to terminate the reactions. Then, 100 µl of 100 mM H_3_PO_4_ was added to each well and mixed thorough using the same mixing program described above to acidify the samples. The 96 well plate was sealed with Sealing Tape and loaded into the HPLC autosampler. Itaconate was measured by HPLC with 10 mM H_3_PO_4_ mobile phase (1 ml/min) and UV detection at 210 nm. Curves of enzyme rate *v* over substrate concentration [S] were fitted using GraphPad Prism 9 with the Michaelis-Menten equation *v* = *k*_cat_ · [S]/(*K*_M_ + [S]) to determine *k*_cat_ and *K*_M_. Each assay was performed in duplicate or triplicate.

### Cell culture

The human myelomonocytic leukaemia cell line THP1 (DSMZ no. ACC 16) was differentiated into adherent macrophages with 125 ng/ml phorbol-12-myristate-13-acetate (Sigma-Aldrich; #P8139) for 48 h and then incubated in fresh medium for an additional 24 h^8^. The resulting differentiated (dTHP1) cells were stimulated with 200 ng/ml LPS (Sigma, #L6511) and 400 U/ml human IFN-γ (PeproTech, #300-02) for 24 h to induce ACOD1 expression.

### Quantification of *cis*-aconitate in THP1 cells

Measurements were performed according to our validated high performance liquid chromatography-tandem mass spectrometry (HPLC-MS/MS) assay^8,30^. The intracellular amounts of *cis*-aconitate were calculated using an external calibration curve with increasing levels of analyte. The protein precipitated during the metabolite extraction was quantified using the BCA protein assay (Thermo Scientific, #23225). The cell number was estimated using a standard curve relating total protein amount to cell number. Assuming that dTHP1 cells are spherical with a cell diameter *d* = 27 μm, the volume *V*_*cell*_ of one cell was calculated as ${V}_{cell}=\frac{4}{3}{\uppi }{\left(\frac{d}{2}\right)}^{3}$^31^. We calculated the intracellular *cis*-aconitate concentration *c* from *n*, the amount of *cis*-aconitate in mol units, using the formula *c* $=\frac{n}{{V}_{cell}\times \text{c}\text{e}\text{l}\text{l}\,\, \text{n}\text{u}\text{m}\text{b}\text{e}\text{r}}$ .

## Electronic supplementary material

Below is the link to the electronic supplementary material.

Supplementary Material 1

Supplementary Material 2

## Data Availability

All data generated or analysed during this study are included in this published article and its supplementary information files.
