# Supplementary material for: Effect of pH and buffer on substrate binding and catalysis by cis-aconitate decarboxylase
Source: Sci Rep. 2025 Feb 11;15:5076. doi: 10.1038/s41598-025-89341-1 (PMC11814083; doi:10.1038/s41598-025-89341-1)
Supplement: Supplementary file 2 — Supplementary Material 2 [file 41598_2025_89341_MOESM2_ESM.pdf]

## Supplementary Information

# Effect of pH and buffer on substrate binding and catalysis by *cis*-aconitate decarboxylase

Mingming Zhao, Chutao Chen, Wulf Blankenfeldt, Frank Pessler, Konrad Büssow

### Figure S1

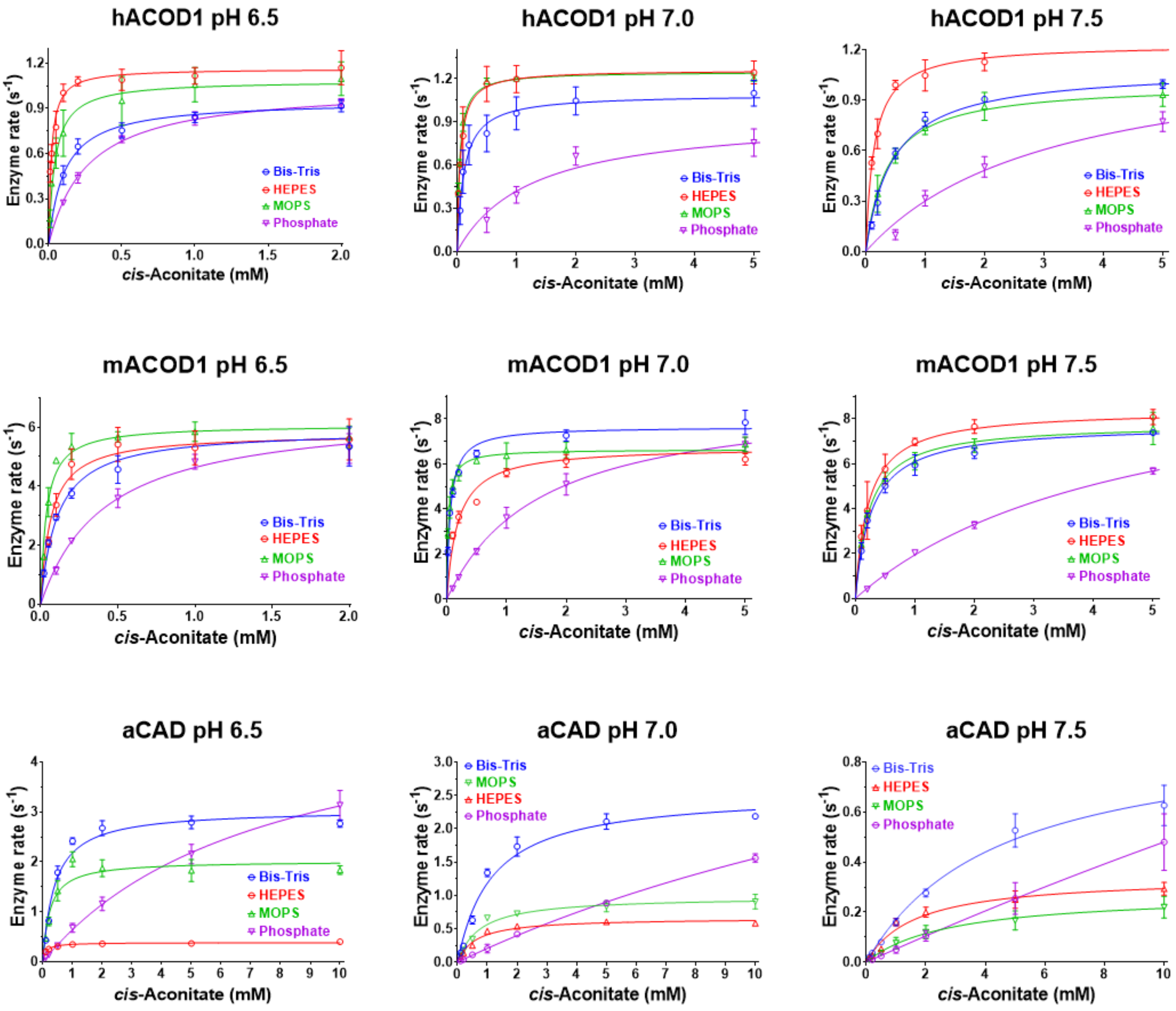


Michaelis-Menten curves of hACOD1, mACOD1, and aCAD using 200 mM of Bis-Tris, HEPES, MOPS and sodium phosphate buffer at pH 6.5, 7.0, 7.5. The resulting kinetic parameters were used for Figure 2.

## Figure S2

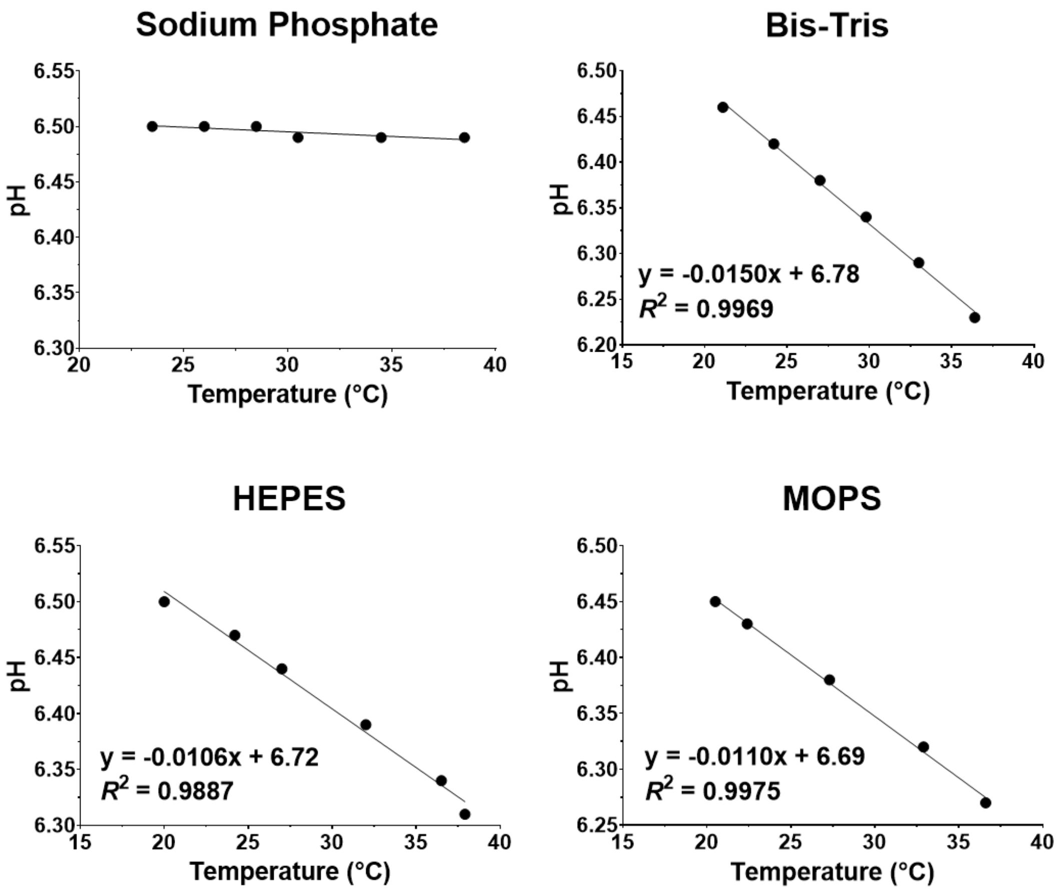


Effect of temperature on pH values of sodium phosphate, Bis-Tris, HEPES and MOPS. The change of pH values of Bis-Tris, HEPES and MOPS with temperature (ΔpH/°C) are linear. Bis-Tris, HEPES, and MOPS exhibit significant pH decreases with rising temperature, with ΔpH/°C values of -0.015, -0.0106, and -0.0110, respectively.

## Figure S3

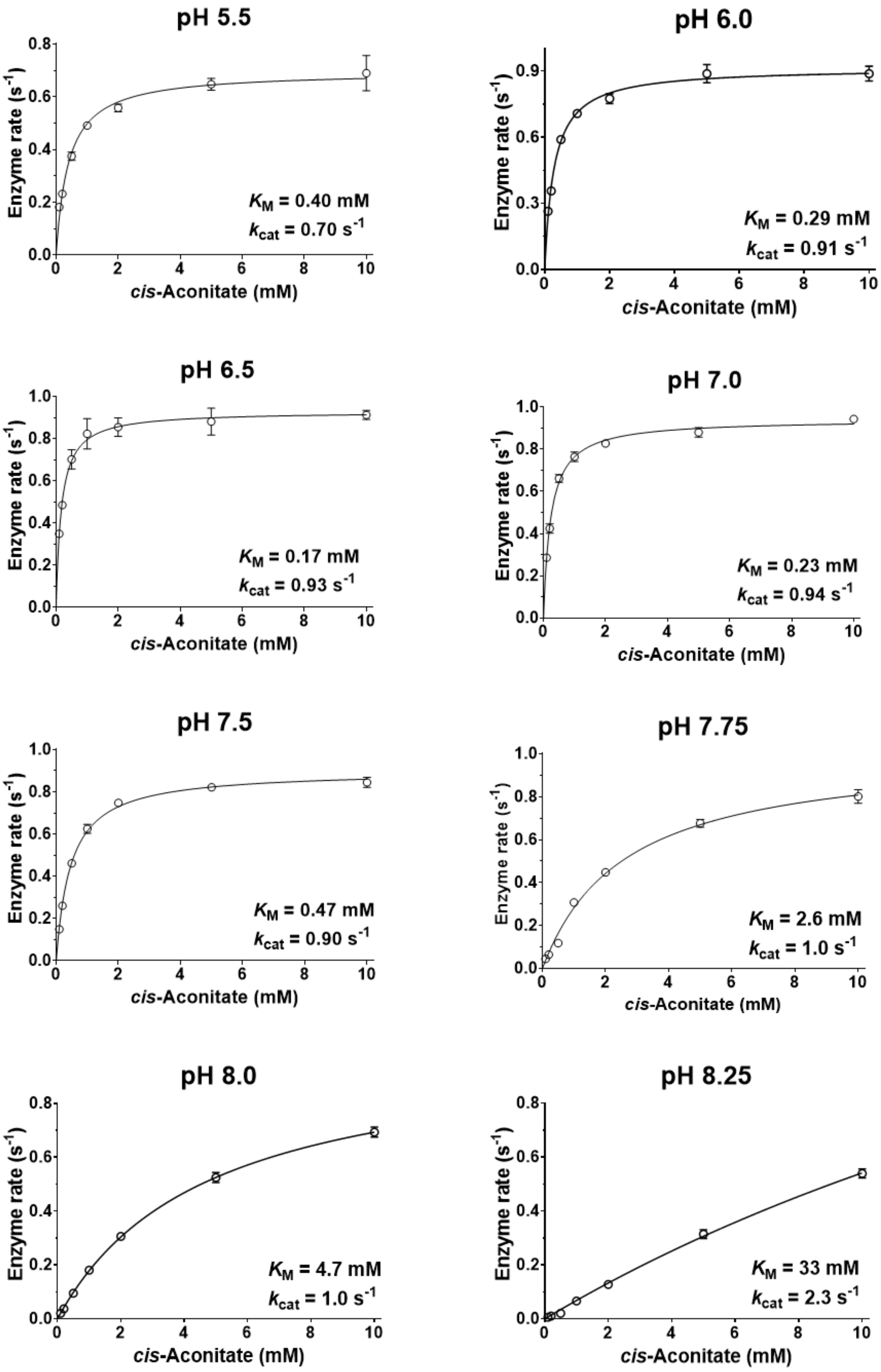


Michaelis-Menten curves of hACOD1 in 50 mM MOPS, 100 mM NaCl at a pH range of 5.5–7.5. The resulting kinetic parameters were used for Figure 4 and Table 1.

## Figure S4

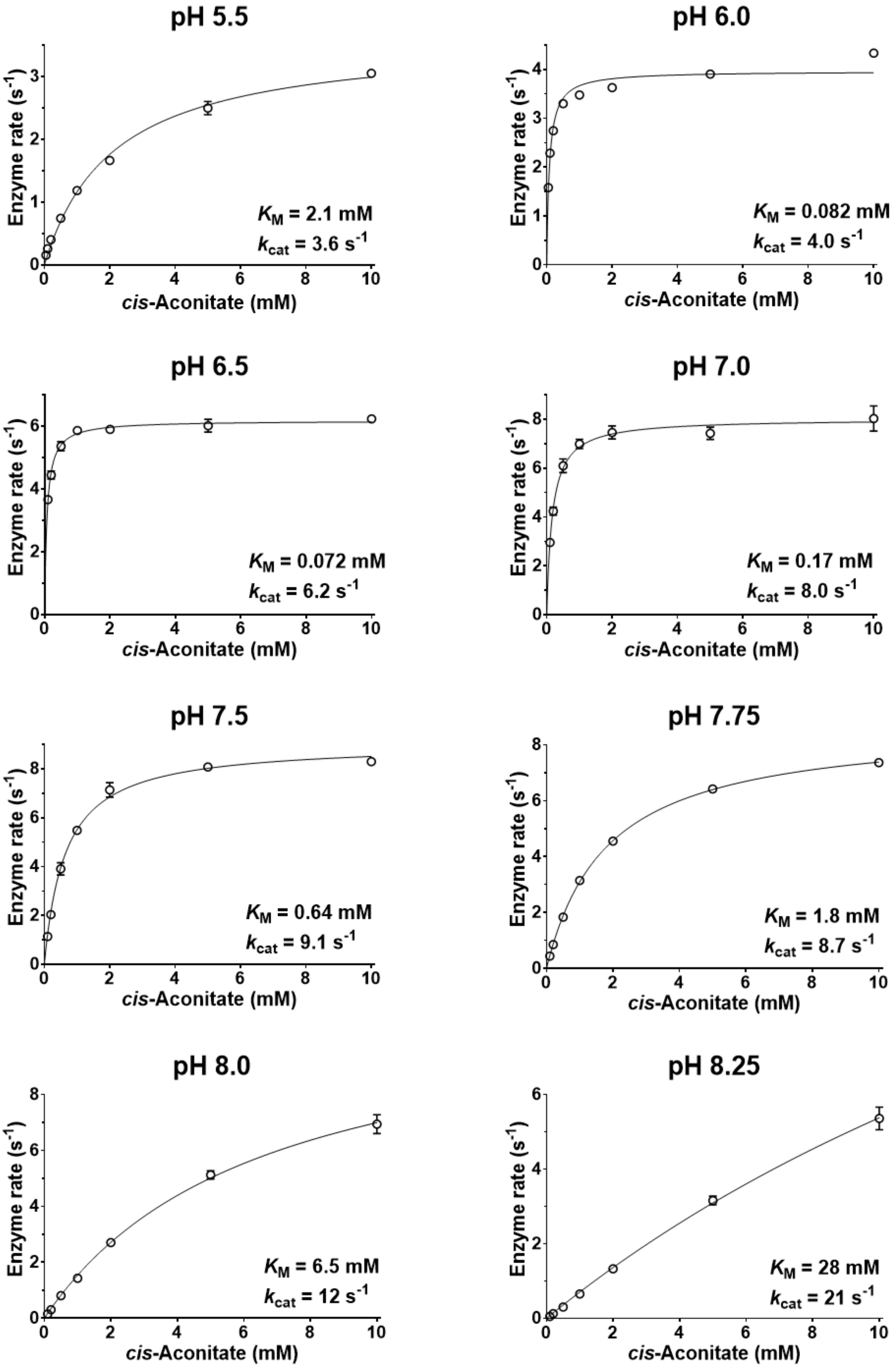


Michaelis-Menten curves of mACOD1in 50 mM MOPS, 100 mM NaCl at a pH range of 5.5–7.5. The resulting kinetic parameters were used for Figure 4 and Table 1.

## Figure S5

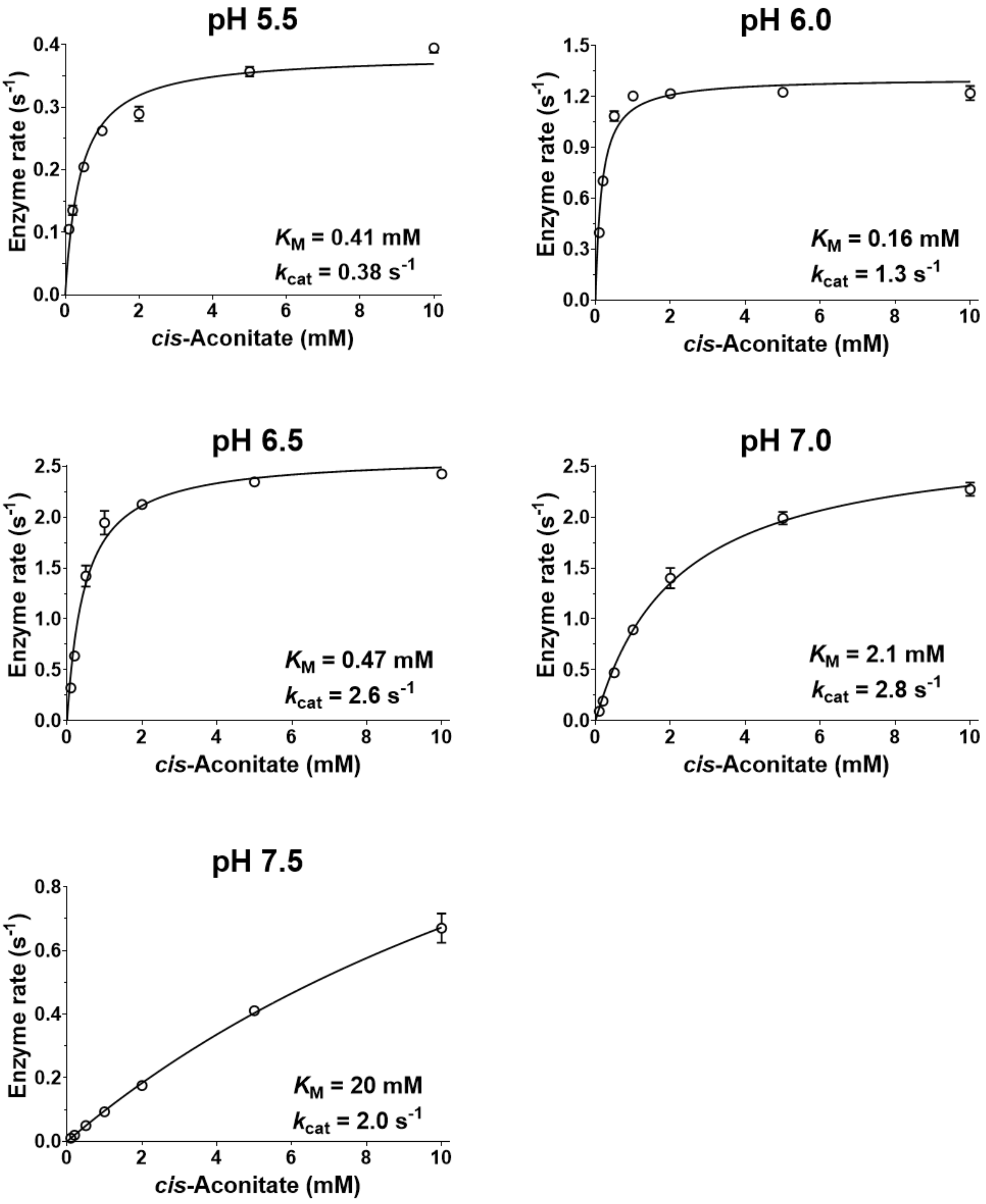


Michaelis-Menten curves of aCAD in 50 mM MOPS, 100 mM NaCl at a pH range of 5.5–7.5. The resulting kinetic parameters were used for Figure 4 and Table 1.

## Figure S6

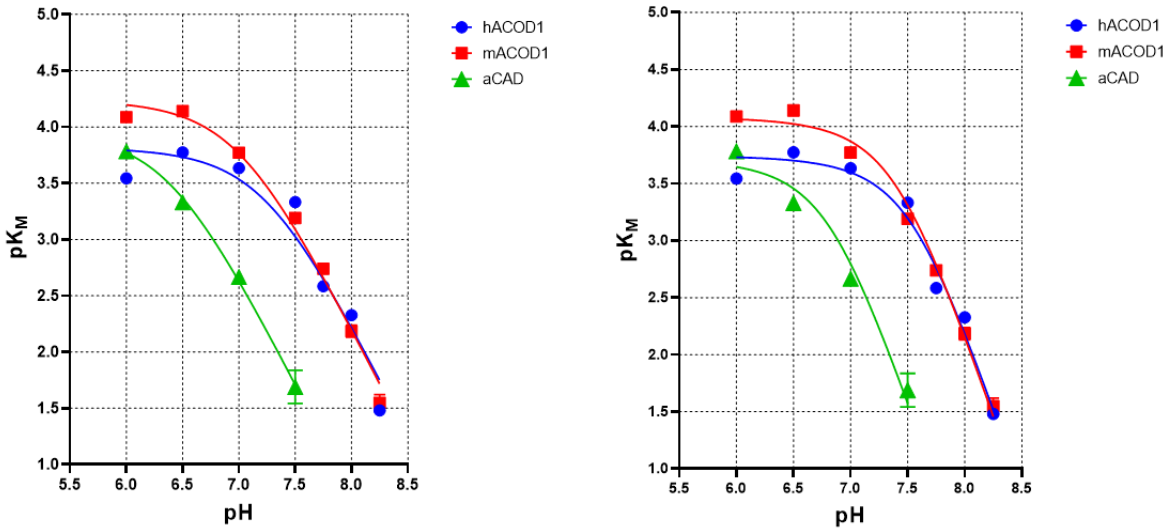


$$\mathrm{E} \underset{K_a}{\overset{\mathrm{H}^+}{\rightleftharpoons}} \mathrm{EH}^{+} \underset{K_a}{\overset{\mathrm{H}^+}{\rightleftharpoons}} \mathrm{EH}_2^{2+} \underset{K_{\mathrm{M0}}}{\overset{\mathrm{S}}{\rightleftharpoons}} \mathrm{EH}_2^{2+}\mathrm{S}$$

$$\mathrm{E} \underset{K_a}{\overset{\mathrm{H}^+}{\rightleftharpoons}} \ldots \underset{K_a}{\overset{\mathrm{H}^+}{\rightleftharpoons}} \mathrm{EH}_3^{3+} \underset{K_{\mathrm{M0}}}{\overset{\mathrm{S}}{\rightleftharpoons}} \mathrm{EH}_3^{3+}\mathrm{S}$$

$$K_M = K_{M0}\left(1 + \frac{K_a}{[\mathrm{H}^+]} + \frac{K_a^2}{[\mathrm{H}^+]^2}\right)$$

$$K_M = K_{M0}\left(1 + \frac{K_a}{[\mathrm{H}^+]} + \frac{K_a^2}{[\mathrm{H}^+]^2} + \frac{K_a^3}{[\mathrm{H}^+]^3}\right)$$

Nonlinear regression of the p$K_{\mathrm{M}}$-pH data of Figure 4B. The data for pH 6.0-8.25 was fitted with two alternative models using GraphPad Prism according to M. Dixon [13]. On the left side, the equation for curve fitting corresponds to two histidine residues that need to be protonated for substrate binding. On the right side, the model corresponds to three histidine residues. E, enzyme, S, substrate. The models were simplified by assuming uniform $K_{\mathrm{a}}$ value for all protonation reactions. $K_{\mathrm{M0}}$ is the $K_{\mathrm{M}}$ value of the enzyme when all active site histidines are protonated. Nonlinear regression resulted in $K_{\mathrm{M0}}$ values of 0.05-0.19 mM and p$K_{\mathrm{a}}$ values of 6.5-7.5. The data are not sufficient to decide which of the two models is correct for the three enzymes. The data could not be fitted with a model corresponding to a single histidine residue.

## Figure S7

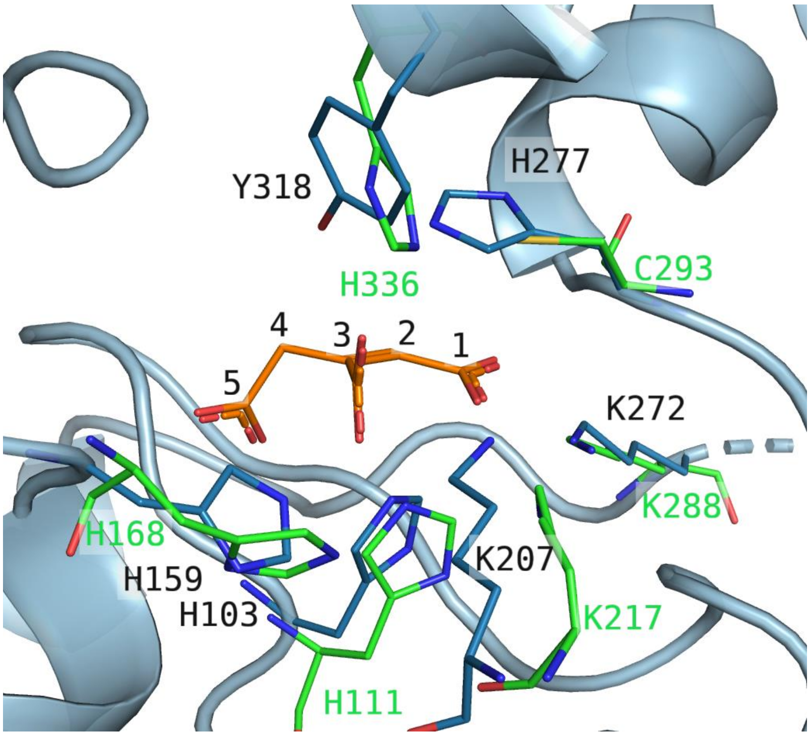


Comparison of aCAD and hACOD1 active sites. The model of the complex of hACOD1 (blue) with *cis*-aconitate (orange) shown in Figure 1B was aligned to a structure of aCAD predicted by AlphaFold2. The aCAD structure was obtained from the AlphaFold Protein Structure Database (structure AF-Q0C8L3-F1-v4 for UniProt Q0C8L3) [14]. aCAD residues are shown in green. His103 and His159 of hACOD1 are conserved in aCAD and correspond to His111 and His168. His277 is not conserved, but His336 of aCAD is located adjacently in the AlphaFold model, so that it can interact with *cis*-aconitate.

## Figure S8

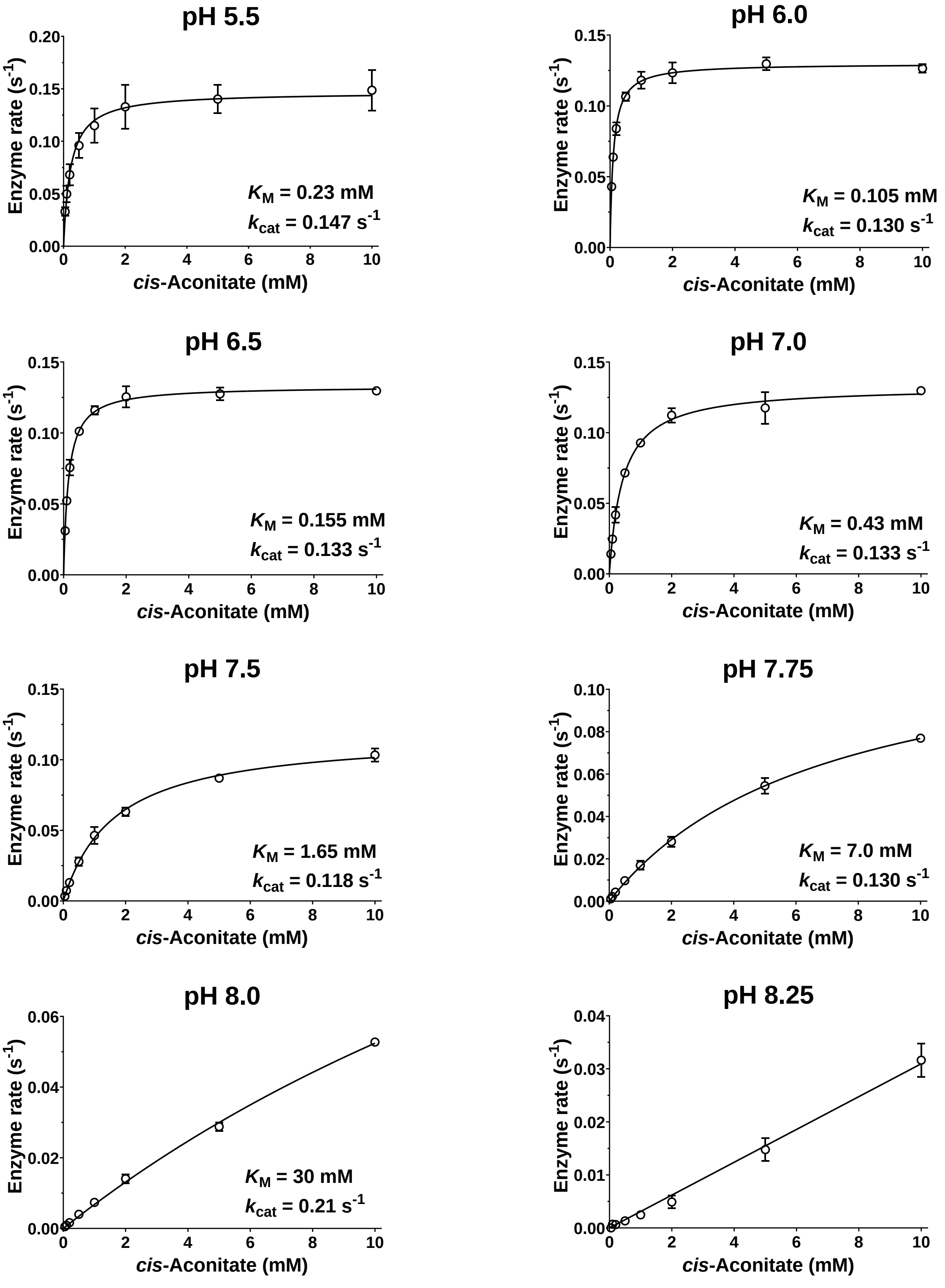


Michaelis-Menten curves of the hACOD1 His159Ala mutant in 50 mM MOPS, 100 mM NaCl at a pH range of 5.5–7.5. The resulting kinetic parameters were used for Figure 4 and Table 1.
